# Celebrities and Medicine: A Potent Combination

**Published:** 2014-03-01

**Authors:** Pamela Hallquist Viale

When it comes to health-related concerns, celebrity commercials and endorsements have always made me a little nervous. The direct-to-consumer advertisements with known personalities such as Bob Dole for erectile dysfunction or Sally Field for bone health often made me cringe. I couldn’t help but wonder if television viewers would really understand whether or not the proposed treatment was right for them. Or would they just be swayed by the celebrity exposure and endorsement? The sticky issue of celebrities being paid for their endorsements really should impact the audience’s outlook as well, but does it? Yet when Angelina Jolie came out with the very frank story of her potential risk for breast and ovarian cancer due to her BRCA1 mutation status, it really got me thinking. The public and the media paid attention too. Health-care professionals, in particular, have debated the positive aspects of her announcement and the subsequent newspaper and media coverage of the "Angelina Jolie effect" and the "Jolie gene." In terms of health care, has this been a positive or negative development?

## The Angelina Jolie Effect

In a May 2013 op-ed report for The New York Times, Ms. Jolie announced her decision to undergo bilateral prophylactic mastectomies. Because of a BRCA1 mutation, her lifetime risk of developing breast cancer was 87%, with a 50% chance of developing ovarian cancer; her mother had died of ovarian cancer at age 56. Ms. Jolie’s surgery reduced her risk to approximately 5% (Hurley, 2013). She "went public" with her mutation status, her cancer risk, and her subsequent decision to undergo surgery instead of keeping it private because she hoped to educate other at-risk women about getting gene-tested and knowing their options. The intense media storm surrounding her decision continues, with demand for the genetic test undoubtedly increased.

## Celebrity Medicine in Health Care

Is the media storm surrounding Ms. Jolie a positive effect for health care and women at risk for the BRCA1 gene mutation? Kamenova, Reshef, and Caulfield published a recent study on the media coverage of Ms. Jolie’s prophylactic surgeries in the journal Genetics in Medicine (2013). Their study examined content analysis of print news, specifically looking at the tone of discussions and how journalists reported on BRCA1/2 mutations and testing as well as hereditary breast and ovarian cancer. The researchers also looked at whether or not concerns were raised about the impact of celebrities on patient choices. The results showed that even though the press did comment on important issues regarding predictive genetic testing and the preventive options for women at high risk for these hereditary cancers, specific key information on the rarity of Jolie’s condition was not adequately reported to the public. Researchers concluded that the media reported Ms. Jolie’s condition and surgical procedures via a significant positive slant without describing the rarity of her specific circumstances. The authors of the study also described the challenge of "celebrity medicine" and how celebrities can influence individuals’ medical decision-making (Kamenova, Reshef, & Caulfield, 2013).

For the relatively small number of at-risk women who were not aware of their personal risk for the BRCA1 gene mutation, genetic testing can certainly make a difference. This information, coupled with a frank discussion with a health-care provider regarding potential treatment options, can be a lifesaver. However, surgery is not always the sole treatment option; close monitoring or the use of tamoxifen and raloxifene may be appropriate courses of action as well (Lee, 2013).

## Opening the Discussion

Whether I feel that celebrity medicine is a positive or negative phenomenon, I do believe that Ms. Jolie’s decision to reveal her personal risk and subsequent surgical procedures was a gutsy one. She certainly wasn’t paid for her disclosure, and one could argue that her revelation might have had a negative outcome considering her career choice. We cannot control the media coverage of her decision, and we cannot expect every media article regarding her case to present a complete, accurate picture of appropriate care in her situation. What I hope, though, is that health-care professionals take every opportunity to use this information to start frank discussions with appropriate patients regarding risk, treatment, and options. The advanced practitioner is in an ideal position to continue that discussion into an honest examination of the facts as pertaining to the individual patient. Genetic testing isn’t appropriate for everyone, but awareness of the possibility of testing shouldn’t be seen as a negative thing.

## JADPRO Live Recap

The first JADPRO Live symposium, which was held January 24 through 26 in St. Petersburg, Florida, was a resounding success! Almost 250 attendees had the opportunity to network with their peers, hear valuable updates on the care of patients with cancer, as well as receive up to 13 hours of continuing education credit. The opening panel discussion with Peter Yu, MD, president-elect of ASCO; Robert W. Carlson, MD, Chief Executive Officer of the NCCN; Louis B. Harrison, former president and former chairman of ASTRO; and Steven Allen, MD, chair of the ASH Committee on Practice set the tone for an incredible 2 days of focused and relevant education for the advanced practitioner (AP). Heather Hylton’s keynote address provided inspiration for what we, as advanced practitioners, do every day while caring for our patients.

Attendees enjoyed the opportunity to share insights and discuss key legislative issues and other practice concerns with our renowned faculty. If you weren’t able to join us, you will be able to access selected content from the conference soon on the JADPRO website. In the meantime, don’t miss the abstracts from our poster session, which appear on pages 144–150 of this issue. Be sure to join us for our next symposium, where we will continue to provide quality education focused on your needs as an advanced practitioner!

## References

[1] Hurley R. (2013). Angelina Jolie’s double mastectomy and the question of who owns our genes.. *British Medical Journal*.

[2] Kamenova K., Reshef  A., Caulfield T. (2013). Angelina Jolie’s faulty gene: Newspaper coverage of a celebrity’s preventive bilateral mastectomy in Canada, the United States, and the United Kingdom. *Genetics in Medicine*.

[3] Lee J. (2013). Celebrity impact. Benefits, risks seen in hype over Jolie’s disclosure.. * Modern Healthcare*.

